# Serum endocan levels in patients with peripheral artery disease: an exploratory cross-sectional study

**DOI:** 10.3389/fcvm.2026.1769347

**Published:** 2026-08-06

**Authors:** Canan Aydoğan, Hüseyin Tezcan, Nazif Aygül, Abdullah Sivrikaya, Bülent Behlül Altunkeser, Abdullah Tunçez, Aslıhan Merve Toprak Su, Kenan Demir, Büşra Ecer

**Affiliations:** 1 Department of Cardiology, Konya Numune Hospital, Konya, Türkiye; 2 Department of Cardiology, Konya Selcuk University Medical Faculty Hospital, Konya, Türkiye; 3 Department of Biochemistry, Konya Selcuk University Medical Faculty Hospital, Konya, Türkiye; 4 Department of Cardiology, Selcuk University Faculty of Medicine, Konya, Türkiye; 5 Department of Cardiology, Kayseri State Hospital, Kayseri, Türkiye; 6 Department of Biochemistry, Konya Beyhekim Education and Research Hospital, Konya, Türkiye

**Keywords:** endocan, endothelial activation, exploratory study, inflammation, peripheral artery disease

## Abstract

**Objectives:**

To explore whether circulating endocan concentrations differ between adults with imaging-confirmed peripheral artery disease (PAD) and controls without clinically evident PAD, and to generate hypotheses regarding the relationship between endocan and PAD-associated endothelial and inflammatory activity.

**Methods:**

In this single-centre exploratory cross-sectional study, 56 patients with imaging-confirmed PAD were compared with 54 cardiovascular risk-enriched controls without clinically evident PAD based on pulse examination and handheld Doppler assessment. Serum endocan was measured by ELISA, and exploratory between-group and multivariable analyses were performed.

**Results:**

The distributions of major cardiovascular comorbidities were statistically comparable between groups; however, the comparator group itself had a substantial cardiovascular-risk burden, including coronary artery disease in 74.1%, diabetes mellitus in 46.3%, hypertension in 59.3%, hyperlipidemia in 83.3%, and smoking in 44.4%. Mean serum endocan concentrations were numerically higher in the PAD group than in the comparator group (218.8 ± 166.9 vs. 170.5 ± 90.0 pg/mL), but the between-group difference did not reach statistical significance (*p* = 0.063). PAD status was not independently associated with serum endocan following multivariable adjustment.

**Conclusions:**

In this exploratory cohort, serum endocan concentrations were numerically higher in patients with PAD, but the between-group difference was not statistically significant and no independent association with PAD was demonstrated after adjustment. Interpretation is materially limited by the cardiovascular risk-enriched comparator population, in whom generalized endothelial activation and possible subclinical PAD may have reduced between-group separation. These findings do not validate endocan as a PAD-specific diagnostic biomarker and cannot be extrapolated to comparisons with low-risk healthy populations. Rather, they generate hypotheses for future studies using uniformly phenotyped healthy and risk-matched comparator groups.

## Introduction

Peripheral artery disease (PAD) is a common manifestation of systemic atherosclerosis and is associated with substantial cardiovascular morbidity and mortality (1). Although established clinical and vascular diagnostic methods remain central to PAD assessment, circulating biomarkers remain of research interest because they may provide insight into endothelial and inflammatory pathways and may identify candidates for subsequent, formally designed diagnostic or prognostic studies (2).

Endothelial cell-specific molecule-1 (ESM-1), also known as **endocan**, is a soluble dermatan sulfate proteoglycan secreted by the vascular endothelium (3). Circulating endocan has been proposed as a marker of endothelial activation and inflammation and has been investigated across a range of cardiovascular conditions linked to endothelial dysfunction (4, 5).

Importantly, emerging PAD-focused data suggest that endocan may also be relevant in lower-extremity atherosclerotic disease. In a clinical PAD cohort, Yazman et al. reported significantly higher endocan concentrations in PAD patients compared with controls and demonstrated a significant reduction in endocan levels at 1-month follow-up after treatment, supporting further investigation of endocan as a correlate of PAD-related endothelial/inflammatory activity and treatment response (6). In a high-risk population of non-dialysis patients with chronic kidney disease (stages 3–5), Cheng et al. found higher endocan levels in individuals with low ABI (<0.9) and identified serum endocan as an independent correlate of ABI-defined PAD in multivariable analysis (7). Conversely, Ağaç et al. reported no significant association between endocan and ABI in asymptomatic hypertensive individuals, suggesting that results may vary by population and disease context and underscoring the need for further studies in well-characterized PAD cohorts (8).

Given the limited and heterogeneous PAD-specific literature, the present study was undertaken as an exploratory, hypothesis-generating investigation. We aimed to compare serum endocan concentrations between adults with imaging-confirmed PAD and controls without clinically evident PAD and to explore whether any observed association persisted after adjustment for major cardiovascular covariates. We did not aim to validate endocan as a diagnostic biomarker, establish a clinical cut-off value, or evaluate diagnostic sensitivity, specificity, or prognostic performance.

## Materials and methods

### Study design

This single-centre, exploratory, hypothesis-generating cross-sectional study was conducted at the Cardiology Clinic of Selcuk University Faculty of Medicine between March and November 2019. A total of 110 adults were enrolled: 56 patients with imaging-confirmed PAD and 54 controls without clinically evident PAD. The investigation was designed to generate preliminary estimates of between-group differences and associations; it was not designed as a diagnostic-accuracy or biomarker-validation study, and no separate derivation or validation cohort was included. The comparator group should not be interpreted as a healthy reference population. Coronary artery disease, diabetes mellitus, hypertension, hyperlipidemia, and smoking were not exclusion criteria for this group. Accordingly, the study compared patients with PAD with a cardiovascular risk-enriched clinical comparator group without clinically evident PAD.

The study was approved by the Selcuk University Faculty of Medicine Ethics Committee (Approval No: 2019/194), and written informed consent was obtained from all participants.

### Inclusion criteria

All participants were required to be ≥18 years of age.

- PAD group: PAD diagnosis was confirmed using imaging modalities employed in routine clinical practice, including invasive peripheral angiography and/or CT or MR angiography, with ultrasonography as clinically indicated.
- Comparator group: Participants without clinically evident PAD based on physical examination and handheld Doppler assessment. A systematic ankle–brachial index measurement or vascular imaging protocol was not applied to all comparator participants. Coronary artery disease and conventional cardiovascular risk factors were not exclusion criteria; therefore, this group represented a cardiovascular risk-enriched clinical comparator population rather than a healthy control population.

### Exclusion criteria

Participants were excluded if they had chronic liver disease, chronic kidney failure, active systemic infection, decompensated heart failure, cancer, inflammatory or rheumatic disease, or a history of acute coronary syndrome within the previous 3 months.

### Demographic and clinical data collection

Demographic and clinical data were recorded at enrollment, including age, sex, cardiovascular risk factors, comorbidities, smoking status, and medication use.

In the PAD group, disease severity staging (Fontaine and Rutherford classifications) and management approach were also recorded.

Medication history was recorded from routine clinical documentation at the time of enrollment. Detailed exposure data (dose, duration, adherence, and timing relative to blood sampling) were not uniformly available; therefore, medications were not analyzed as independent predictors of serum endocan in this cross-sectional study.

### Biochemical analysis

Laboratory indices were obtained and recorded as part of the baseline evaluation, including lipid parameters (LDL, HDL, triglycerides), renal function (serum creatinine), and complete blood count parameters (hemoglobin, neutrophil count, lymphocyte count). The neutrophil-to-lymphocyte ratio (NLR) was calculated as neutrophil count divided by lymphocyte count.

### ELISA methodology

Venous blood samples were collected from all participants at the time of enrollment into serum tubes. After clot formation, the samples were centrifuged to separate serum, and the serum aliquots were stored under frozen conditions until analysis. Serum endocan concentrations were measured using an enzyme-linked immunosorbent assay (ELISA) according to the manufacturer's instructions and expressed in pg/mL.

### Sample size consideration

Because PAD-specific endocan effect estimates in imaging-confirmed cohorts were limited at the time of study planning, this study was designed as an exploratory cross-sectional investigation with consecutive enrolment over a predefined period rather than as a formal biomarker-validation study. The intended sample size was approximately 50–60 participants per group. With 56 and 54 participants, the study had approximately 80% power, at a two-sided *α* level of 0.05, to detect a moderate standardized between-group difference of approximately Cohen's *d* = 0.54. However, the study was not powered to detect small effects or to estimate diagnostic cut-off values, diagnostic-accuracy measures, or clinical performance with sufficient precision.

### Statistical analysis

Data were analyzed using SPSS version 21. Continuous variables were expressed as mean ± standard deviation, and categorical variables were presented as frequencies and percentages. Categorical variables were compared using the chi-square test. Continuous variables were compared using Student's *t*-test or the Mann–Whitney *U* test, as appropriate according to the distribution of the data.

The principal exploratory comparison was the difference in serum endocan concentrations between the PAD and control groups. To explore whether PAD status was independently associated with serum endocan, a parsimonious multivariable linear regression analysis was performed with endocan as the dependent variable and PAD status as the main explanatory variable. Covariates were selected *a priori* on the basis of clinical relevance and included age, sex, diabetes mellitus, hypertension, hyperlipidemia, smoking status, coronary artery disease, and serum creatinine.

All analyses were considered exploratory and hypothesis-generating. A two-sided *p*-value <0.05 was considered statistically significant; however, the findings were interpreted according to the direction, magnitude, and uncertainty of the observed effects and were not considered evidence of biomarker validation. No ROC-curve analysis, AUC estimation, sensitivity or specificity assessment, cut-off optimization, calibration analysis, or internal or external validation was performed because diagnostic performance was outside the scope of the study.

No adjustment for multiple comparisons was applied to the secondary laboratory analyses; therefore, these findings should be interpreted as exploratory.

## Results

The study included 110 patients aged between 35 and 81 years, divided into two groups: 56 patients with PAD and 54 controls without PAD. Baseline demographic and clinical characteristics are presented in Table 1. The PAD and control groups were similar in terms of age (62.5 ± 9.3 vs. 62.1 ± 8.8 years; *p* = 0.84) and gender distribution (80.4% male in PAD vs. 79.6% male in control; *p* = 0.84). The PAD and comparator groups were similar in age and sex distribution. There were no statistically significant between-group differences in coronary artery disease, diabetes mellitus, hypertension, hyperlipidemia, or smoking status. Nevertheless, the absolute cardiovascular-risk burden in the comparator group was substantial: 74.1% had coronary artery disease, 46.3% had diabetes mellitus, 59.3% had hypertension, 83.3% had hyperlipidemia, and 44.4% had smoking exposure. Thus, the comparator group represented a cardiovascular risk-enriched clinical population rather than a healthy reference population.

**Table 1:** Baseline characteristics of the PAD and cardiovascular risk-enriched comparator groups.

Characteristic	PAD group (*n* = 56)	Control group (*n* = 54)	*p*-value
Age (years)	62.5 ± 9.3	62.1 ± 8.8	0.84
Male, *n* (%)	45 (80.4%)	43 (79.6%)	0.84
CAD, *n* (%)	40 (71.4%)	40 (74.1%)	0.75
DM, *n* (%)	27 (48.2%)	25 (46.3%)	0.84
HT, *n* (%)	33 (58.9%)	32 (59.3%)	0.97
Smoking, *n* (%)	27 (48.2%)	24 (44.4%)	0.69
HL, *n* (%)	48 (85.7%)	45 (83.3%)	0.73
SBP (mmHg)	119 ± 14.9	120 ± 11.4	0.88
DBP (mmHg)	73 ± 7.6	72 ± 5.9	0.43

The comparator group consisted of participants without clinically evident PAD based on physical examination and handheld Doppler assessment. It was not a healthy reference population, because established coronary artery disease and conventional cardiovascular risk factors were not exclusion criteria.

CAD, coronary artery disease; DM, diabetes mellitus; HT, hypertension; HL, hyperlipidemia; SBP, systolic blood pressure; DBP, diastolic blood pressure; PAD, peripheral artery disease.

Treatment modalities for PAD patients are summarized in Table 2. Of the 56 patients with PAD, the majority (73.2%) underwent percutaneous intervention, 19.6% received surgical treatment, and 7.1% were managed with medical therapy alone.

**Table 2:** Treatment modalities in PAD patients.

Treatment method	Patients (*n* = 56)
Medical therapy	4 (7.1%)
Percutaneous intervention	41 (73.2%)
Surgical intervention	11 (19.6%)

Table 3 shows the distribution of patients with PAD according to the Fontaine and Rutherford classifications. In the Fontaine classification, 48.2% of patients were at stage IIb, whereas 17.9% were at stage IV. According to the Rutherford classification, 44.6% were in category 2 and 21.4% were in category 4, indicating a broad range of PAD severity among participants.

**Table 3:** Fontaine and Rutherford classifications of PAD patients.

Fontaine stage	Patients (%)	Rutherford stage	Category	Patients (%)
I	0	0	0	0
IIa	7 (12.5%)	I	1	7 (12.5%)
IIb	27 (48.2%)	I	2	25 (44.6%)
		I	3	2 (3.6%)
III	7 (12.5%)	II	4	12 (21.4%)
IV	10 (17.9%)	III	5	6 (10.7%)
		III	6	4 (7.1%)

Laboratory parameters are summarized in Table 4. Compared with controls, patients with PAD had lower HDL levels (36.28 ± 9.0 vs. 42.8 ± 9.39 mg/dL; *p* < 0.001) and higher creatinine levels (0.97 ± 0.25 vs. 0.84 ± 0.18 mg/dL; *p* = 0.003). Hemoglobin levels were also significantly lower in the PAD group (12.46 ± 2.13 vs. 14.08 ± 1.63 g/dL; *p* < 0.001). In addition, the PAD group had higher neutrophil counts (5.77 ± 1.90 vs. 4.94 ± 1.81 × 10^9^/L; *p* = 0.022) and a higher neutrophil-to-lymphocyte ratio (3.54 ± 1.93 vs. 2.57 ± 1.47; *p* = 0.004). Overall, compared with controls, PAD patients demonstrated a more adverse inflammatory/clinical laboratory profile (lower HDL, higher creatinine, lower hemoglobin, higher neutrophil count and higher NLR; all *p* < 0.05), while serum endocan was numerically higher but did not reach statistical significance (*p* = 0.063).

**Table 4:** Laboratory parameters in PAD and control groups.

Parameter	PAD group (*n* = 56)	Control group (*n* = 54)	*p*-value
LDL (mg/dL)	95.5 ± 37.5	95.1 ± 35.9	0.95
TG (mg/dL)	173.75 ± 117.4	155.85 ± 80.3	0.35
HDL (mg/dL)	36.28 ± 9.0	42.8 ± 9.39	<0.001
Cr (mg/dL)	0.97 ± 0.25	0.84 ± 0.18	0.003
EF (%)	49.2 ± 11.6	52.1 ± 8.3	0.142
Endocan (pg/mL)	218.76 ± 166.9	170.48 ± 90.0	0.063
Hb (g/dL)	12.46 ± 2.13	14.08 ± 1.63	< 0.001
Neutrophils (×10^9^/L)	5.77 ± 1.90	4.94 ± 1.81	0.022
Lymphocytes (×10^9^/L)	1.89 ± 0.66	2.15 ± 0.77	0.057
NLR	3.54 ± 1.93	2.57 ± 1.47	0.004

LDL, low-density lipoprotein; TG, triglycerides; HDL, high-density lipoprotein; Cr, creatinine; EF, ejection fraction; Hb, hemoglobin; NLR, neutrophil-to-lymphocyte ratio; PAD, peripheral artery disease.

Serum endocan concentrations were numerically higher in the PAD group than in the control group (218.76 ± 166.9 vs. 170.48 ± 90.0 pg/mL), corresponding to an unadjusted mean difference of 48.28 pg/mL. However, substantial interindividual variability was observed, and the between-group difference did not reach statistical significance (*p* = 0.063). In the exploratory multivariable model adjusting for age, sex, diabetes mellitus, hypertension, hyperlipidemia, smoking status, coronary artery disease, and serum creatinine, PAD status was not independently associated with serum endocan.

## Discussion

This exploratory cross-sectional study compared serum endocan concentrations between 56 patients with imaging-confirmed peripheral artery disease and 54 cardiovascular risk-enriched comparator participants without clinically evident PAD. Serum endocan concentrations were numerically higher in the PAD group; however, the between-group difference was not statistically significant. PAD status was also not independently associated with serum endocan after adjustment for prespecified cardiovascular covariates. Accordingly, these findings should be interpreted as preliminary, hypothesis-generating estimates rather than evidence of a PAD-specific association or validated biomarker performance.

Endocan is an endothelium-derived soluble proteoglycan involved in endothelial activation and inflammatory signaling (3–5). Its expression is influenced by proinflammatory cytokines and angiogenic pathways, and it may participate in leukocyte–endothelium interactions and changes in vascular permeability (9–11). Associations reported in acute myocardial infarction, coronary slow flow, and coronary collateral development further suggest that endocan reflects vascular activity across several cardiovascular settings rather than lower-extremity PAD alone (12–15). This broad biological profile provides a rationale for investigating endocan in atherosclerotic disease but also limits assumptions regarding its specificity for PAD.

PAD-specific evidence remains limited and heterogeneous. Yazman et al. reported higher circulating endocan concentrations in patients with PAD and a reduction following treatment (6). Cheng et al. found an independent association between serum endocan and ABI-defined PAD in patients with stage 3–5 chronic kidney disease (7). In contrast, Ağaç et al. found no significant association between endocan and ABI in asymptomatic individuals with hypertension (8). Differences in PAD definition, disease severity, renal function, background atherosclerotic burden, comparator selection, medication exposure, and laboratory methods may contribute to the variation among studies. The present findings do not reproduce the statistically significant association reported in some previous cohorts but provide additional data from patients with imaging-confirmed PAD and a clinically complex comparator population.

The cardiovascular risk profile of the comparator group is central to the interpretation of our findings. Although clinically evident PAD was not identified using the study assessment, the comparator group had a high prevalence of coronary artery disease, diabetes mellitus, hypertension, hyperlipidemia, and smoking. These conditions may increase background endothelial activation and circulating endocan concentrations, thereby reducing the biological contrast between groups. In addition, PAD was not excluded in all comparator participants using a uniform ankle–brachial index or vascular imaging protocol. Asymptomatic or subclinical PAD therefore cannot be ruled out, and any resulting disease-status misclassification may have further attenuated the observed difference. The nonsignificant comparison may consequently reflect limited PAD specificity of endocan, a comparator-spectrum effect, residual disease misclassification, limited statistical precision, or a combination of these factors. The present design cannot distinguish among these explanations.

Patients with PAD also had higher neutrophil counts and neutrophil-to-lymphocyte ratios and lower HDL cholesterol and hemoglobin levels than comparator participants. These findings are consistent with a less favorable inflammatory and clinical profile. Although an elevated neutrophil-to-lymphocyte ratio has previously been associated with PAD severity and outcomes (16), the present study did not evaluate associations between these laboratory variables and PAD severity, treatment response, or prognosis. These secondary comparisons should therefore be considered exploratory and should not be interpreted as evidence of biomarker performance.

The absence of an independent association between PAD status and serum endocan after multivariable adjustment further supports a cautious interpretation. Nevertheless, the modest sample size and the possibility of residual confounding prevent the adjusted analysis from establishing the absence of a smaller association. Importantly, the study did not evaluate ROC curves, area under the curve, sensitivity, specificity, calibration, diagnostic thresholds, prognostic outcomes, or incremental value beyond established clinical and vascular assessments. No conclusion regarding diagnostic or prognostic utility can therefore be drawn from the present data. Established clinical assessment, ankle–brachial index measurement, and vascular imaging remain the basis of PAD diagnosis and management (14, 17).

Future studies should include larger, prospectively assembled multicenter cohorts and two clearly defined comparator groups: low-risk healthy participants and cardiovascular risk-matched participants in whom PAD is excluded using a uniform protocol. Standardized ankle–brachial index measurement, supplemented by toe–brachial index or vascular imaging when appropriate, would reduce disease-status misclassification. Prespecified and adequately powered analyses could then determine whether circulating endocan primarily reflects generalized vascular inflammation or has an independent association with PAD. Until such evidence is available, endocan should be considered a candidate for further investigation rather than a clinically validated PAD biomarker.

## Conclusion

In this exploratory cross-sectional study, serum endocan concentrations were numerically higher in patients with PAD than in comparator participants, but the between-group difference was not statistically significant and PAD status was not independently associated with endocan after multivariable adjustment. Interpretation is materially constrained by the cardiovascular risk-enriched comparator population, which may have had increased background endothelial activation, and by the absence of uniform ABI or imaging-based PAD exclusion among comparator participants. These factors may have attenuated between-group separation. Accordingly, the present findings do not validate endocan as a PAD-specific biomarker, cannot establish diagnostic discrimination, and should not be extrapolated to comparisons with low-risk healthy populations. Rather, they generate a hypothesis that should be tested in larger prospective studies including both healthy and cardiovascular risk-matched, uniformly phenotyped comparator groups.

## Limitations

The principal limitation was the use of a cardiovascular risk-enriched comparator group rather than a low-risk healthy reference population. Generalized atherosclerosis and cardiometabolic risk factors may have increased background endothelial activation and reduced the observed contrast in serum endocan concentrations. In addition, PAD was confirmed by vascular imaging in the PAD group but was excluded in comparator participants by physical examination and handheld Doppler assessment rather than by a uniform ankle–brachial index or imaging protocol. Asymptomatic or subclinical PAD therefore cannot be completely excluded.

The modest sample size limited statistical precision and the ability to detect small associations. The cross-sectional design precluded causal, temporal, and prognostic inference, and the single-center, predominantly male population may limit generalizability. Detailed medication dose, duration, adherence, and timing relative to blood sampling were not uniformly available; residual confounding by treatment exposure therefore remains possible. Finally, the study was exploratory and did not evaluate diagnostic accuracy, clinically applicable thresholds, or internal or external validation. The findings should not be used for clinical decision-making.

## Data Availability

The raw data supporting the conclusions of this article will be made available by the authors, without undue reservation.
